# Genome-Wide Identification of circRNAs in Pathogenic Basidiomycetous Yeast *Cryptococcus neoformans* Suggests Conserved circRNA Host Genes over Kingdoms

**DOI:** 10.3390/genes9030118

**Published:** 2018-02-26

**Authors:** Liang Huo, Ping Zhang, Chenxi Li, Kashif Rahim, Xiaoran Hao, Biyun Xiang, Xudong Zhu

**Affiliations:** Beijing Key Laboratory of Genetic Engineering Drug and Biotechnology, Institute of Biochemistry and Molecular Biology, College of Life Sciences, Beijing Normal University (CLS-BNU), Beijing 100875, China; cryptoleon@gmail.com (L.H.); zp1516@163.com (P.Z.); lcx1219@163.com (C.L.); kashifbangash073@gmail.com (K.R.); hxrr_563@163.com (X.H.); xby6024@126.com (B.X.)

**Keywords:** *Cryptococcus neoformans*, next-generation sequencing (NGS), circRNAs, RNA debranching enzyme, GTPase-encoding gene

## Abstract

Circular RNAs (circRNAs), a novel class of ubiquitous and intriguing noncoding RNA, have been found in a number of eukaryotes but not yet basidiomycetes. In this study, we identified 73 circRNAs from 39.28 million filtered RNA reads from the basidiomycete *Cryptococcus neoformans* JEC21 using next-generation sequencing (NGS) and the bioinformatics tool circular RNA identification (CIRI). Furthermore, mapping of newly found circRNAs to the genome showed that 73.97% of the circRNAs originated from exonic regions, whereas 20.55% were from intergenic regions and 5.48% were from intronic regions. Enrichment analysis of circRNA host genes was conducted based on the Gene Ontology and Kyoto Encyclopedia of Genes and Genomes pathway databases. The results reveal that host genes are mainly responsible for primary metabolism and, interestingly, ribosomal protein production. Furthermore, we uncovered a high-level circRNA that was a transcript from the guanosine triphosphate (GTP)ase gene *CNM01190* (gene ID: 3255052) in our yeast. Coincidentally, *YPT5*, *CNM01190*′s ortholog of the GTPase in *Schizosaccharomyces pombe*, protists, and humans, has already been proven to generate circRNAs. Additionally, overexpression of RNA debranching enzyme *DBR1* had varied influence on the expression of circRNAs, indicating that multiple circRNA biosynthesis pathways exist in *C. neoformans*. Our study provides evidence for the existence of stable circRNAs in the opportunistic human pathogen *C. neoformans* and raises a question regarding their role related to pathogenesis in this yeast.

## 1. Introduction

Circular RNAs (circRNAs), characterized by a closed loop structure, have been a hot topic of research in RNA biology since their wildly diverse and multiple functions were confirmed [1,2]. The fact that the 3′ and 5′ ends of those RNAs are joined by covalent bonds makes them lack polyadenylated tails and 5′–3′ polarity [3]. As a result, circRNAs are considered more stable than linear RNA molecules and more resistant to degradation by RNase R, which is an efficient 3′ to 5′ exoribonuclease [4]. 

Although the first case of circRNAs was reported in plant-based virus as early as 1976 [5], circRNAs have been discarded as “junk-RNA developed by messenger RNA (mRNA) splicing” and ignored by most research groups [6]. This situation remained for decades, until abundant circRNAs were uncovered in a variety of normal and malignant human cells in 2012 [7] and circRNAs were demonstrated as efficient “microRNA sponges” in 2013 [8]. With the development of high-throughput sequencing technology and RNA circularization prediction algorithms, such as circular RNA identification (CIRI) [9], circular (CIRC) explorer [10], and known and novel isoform explorer (KNIFE) [11], an increasing number of circRNAs have been detected in protists, yeasts, plants, flies, and mammals [12,13]. 

The mechanism of circRNA biogenesis is intricate, regulated by multiple factors, and varying among different species. In general, the circularization of RNAs can be accomplished through at least four disparate paths: spliceosome-dependent [14], intron-pairing-driven [15], protein factors-associated [16], and lariat-driven paths [17]. Recent studies also reveal distinctly crucial functions of circRNAs, such as microRNA (miRNA) sponge, post-transcription regulation, rolling circle translation, and creation of circRNA-derived pseudogenes [6,18,19]. However, the mechanism has not been illustrated thoroughly and it deserves to be investigated further. 

The basidiomycetous yeast *Cryptococcus neoformans* is an opportunistic human pathogen that has been life-threatening to immunodeficient groups such as human immunodeficiency virus (HIV)-infected patients [20]. Efforts have been made by laboratories worldwide to understand the fundamentals of its pathogenic progress and its virulence determinants. Considering the fact that knowledge about circRNA molecules is limited, it may be necessary to define the potential role of circRNA in *C. neoformans*. Unfortunately, circRNAs have not been reported in this fungus, nor in the whole group of basidiomycetes. Thus, here we attempted to identify circRNAs from *C. neoformans*, and subsequently analyzed the features and conducted functional annotation of those circRNAs. We identified in this study 73 unique circRNAs in this basidiomycetous yeast. Interestingly, we also found the existence of small guanosine triphosphatase (GTPase)-encoding genes, which are conserved circRNA-host genes in yeasts and some other eukaryotic organisms. Finally, we demonstrate the influence of an RNA debranching enzyme, Dbr1, on the expression of circRNAs. 

## 2. Materials and Methods

### 2.1. Strains and Media

The strain *C. neoformans* var. *neoformans* JEC21 (serotype D, MATα) was used for circRNA analysis in this study. Yeast extract–peptone–dextrose (YPD) medium (2% glucose, 2% peptone, 1% yeast extract, pH 6.0) was used for routine growth of *C. neoformans*.

### 2.2. RNA Isolation and Quality Control 

JEC21 was cultured in 5 mL liquid YPD medium for 18 h at 30 °C. Fresh yeast cells were collected by centrifugation, and approximately 0.1 g of yeast cells was washed by wash buffer (0.1 M ethylenediaminetetraacetic acid (EDTA), 0.5 M sodium chloride) three times at 4 °C. Fungal capsule was broken by Bullet Blender Storm 24 (Next Advance, Troy, NY, USA) for 2 min. Total RNA was extracted using the RNAiso (Takara, Shiga, Japan) according to the protocol supplied with the reagent. RNA concentration was measured by POLARstar Omega (BMG Labtech, Offenburg, Germany), and RNA quality was tested by Agilent 2100 (Agilent Technologies, Santa Clara, CA, USA). The quality control threshold was set as follows: A260/A280 ratio > 1.8, A260/A230 ratio > 1.8, RNA integrity number value > 7.0.

### 2.3. Deep RNA Sequencing and In Silico Discovery of Circular RNAs

Construction of a RNA library, as well as deep RNA sequencing, was accomplished by a commercial service (Genewiz, Suzhou, China). Briefly, Ribominus^TM^ transcriptome isolation kit (Thermo Fisher Scientific, Waltham, MA, USA) was used to remove ribosome RNA in the total RNA. RNase R (Takara) treatment was performed according to the manufacturer’s protocol to remove linear RNA in the RNA samples. KAPA Stranded mRNA-seq Kit (Kapa Biosystems, Wilmington, MA, USA) was utilized for the generation of RNA-sequencing (RNA-seq) libraries according to the manufacturer’s protocol. Next-generation sequencing was then conducted on a HighSeqTM 2500 system (Illumina, San Diego, CA, USA). To remove the low-quality reads in the raw paired-end data, such as the primer/adaptor sequences and non-ATGC reads, IlluQC_PRLL.pl v 2.3.3 software [21] was used to perform quality check with the parameter set as 20. The reads with more than or equal to the specified quality score (20 in this study) are filtered as high-quality reads. Subsequently, clean data were aligned to the *C. neoformans* JEC21 genome (Cryptococcus_neoformans.GCA_000091045.1. dna.toplevel.fa, release 37) using Burrows–Wheeler aligner (BWA) (version 0.6) software with default settings [22]. The 19Mb genome sequence of *C. neoformans* JEC21 consists of 14 chromosomes with different lengths changing from 762 kilobase (kb) pairs to 2.3 megabase (Mb) pairs. 

The CIRI algorithm (version 1.2) was the tool to identify circRNAs in *C. neoformans* JEC21 [9]. CIRI was performed with default options, with the computer command: CIRI_v1.2.pl -I input.sam -O output_circRNAs.txt –F genome.fa -P -A Ensembl_Cn37.gtf. Counts of identified circRNA reads were normalized by read length, and the number of reads mapping (spliced reads per billion mapping) was determined after CIRI prediction [23].

### 2.4. Gene Ontology Category and Kyoto Encyclopedia of Genes and Genomes Pathway Analysis

The circRNA host genes were functionally analyzed according to gene ontology (GO) by the database for annotation, visualization, and integrated discovery (DAVID) 6.7 web server (https://david.ncifcrf.gov) with the default options [24]. The KOBAS 2.0 web server was used to uncover the Kyoto Encyclopedia of Genes and Genomes (KEGG) biological pathways of circRNA host genes with the default settings [25]. 

### 2.5. Validation of Circular RNAs

To confirm the existence of certain circRNAs of interest, e.g., circCNYPT5, we adopted an approach of outward polymerase chain reaction (PCR) with a pair of primers designed for outward amplification. Briefly, total RNA was extracted with RNAiso reagent (Takara) as descripted in Section 2.2. complementary DNA (cDNA) synthesis was performed with the FastQuant RT Kit with genomic DNase (gDNase) (Tiangen Biotech, Beijing, China). The 50 μL amplification reaction system contained 0.5 μL Takara Ex Taq, 5 μL 10× Ex Taq Buffer, 4 μL deoxyribonucleotide triphosphates (dNTPs), 2 μL/2 μL forward/reverse primers, and 36.5 μL double distilled water (ddH_2_O). The PCR program was set as follows: 98 °C for 2 min, 32 cycles at 98 °C for 10 s, 55 °C for 20 s, and 72 °C for 30 s; the final elongation step was run at 72 °C for 5 min. PCR products with expected length (~250 base pairs (bp)) were separated by 0.8% agarose gel electrophoresis and purified with TIANgel midi purification kit (Tiangen Biotech) according to the manufacturer’s instruction. Sanger sequencing was employed to confirm the existence of the back-splicing junction sites (Genewiz). 

### 2.6. Other Online Database and Software

The annotation and nomenclature of *C. neoformans* JEC21 genes in this article were referred to the Ensemble Fungi database (http://fungi.ensembl.org). The multiple alignments of amino acid sequences were conducted by the Clustal Omega web server [26] with default settings. 

### 2.7. Construction of DBR1 Gene Overexpression Vector 

To investigate the regulation of circRNAs by *DBR1*, we overexpressed the gene in the wild-type JEC21 strain. The whole *DBR1* gene, including an 800-bp flanking sequence, was obtained by PCR with the protocol described in Section 2.5, except the elongation time for each cycle was 2 min. The pBS-HYG plasmid was linearized with the restriction enzyme Hind III. Then the In-Fusion^®^ HD cloning kit (Takara) was employed to ligate the linearized plasmid and *DBR1* fragment. Subsequently, recombinant plasmid pBS-HYG-DBR1 was linearized by Xba I enzyme and transformed into the wild-type *C. neoformans* JEC21 cells. To select positive transformants, cells were screened on YPD plates containing 100 μg/mL hygromycin. Genomic DNA of two randomly selected clones, OE-1 and OE-2, was extracted and used in subsequent experiments. PCR was performed to confirm the existence of pBS-HYG-DBR1 in the genome of selected transformants using the same protocol as described in Section 2.5.

### 2.8. Quantitative and Semiquantitative Reverse Transcription Polymerase Chain Reaction

Total RNA of JEC21, OE-1, and OE-2 was extracted as described in Section 2.2. Reverse transcription (RT) of total RNA was conducted by Fast Quant RT kit with gDNase (Tiangen Biotech). Briefly, 1 μL total RNA, 2 μL 5× *g* DNA buffer, and 7 μL ddH_2_O were incubated at 42 °C for 10 min, then 2 μL 10× Fast RT Buffer, 1 μL RT enzyme mix, 2 μL Fast Quant RT primer mix, and 5 RNase-Free ddH_2_O were added to previous tubes and incubated at 42 °C for 15 min. The reaction was stopped by incubating at 95 °C for 10 s. For *DBR1* mRNA quantification, LightCycler 480 II and corresponding LC 480 SYBR Green I Master (Roche, Basel, Switzerland) were employed. The PCR reaction system included 10 μL 2× Master Mix, 1 μL forward/reverse primers (10 μm), 1 μL cDNA, and 7 μL ddH_2_O. Each reaction was performed in triplicate. Non-RT RNA was used as a template in negative control and actin mRNA served as reference. Specificity of primers was validated by checking the melting curves. The 2^−ΔΔCt^ method was employed to calculate expression levels of target genes in this study. Semiquantitative reverse transcription PCR was performed using the PCR protocol described in Section 2.5, except only 25 circles were applied. 

## 3. Results

### 3.1. Genomewide Identification of Circular RNAs

To investigate circRNAs on a genome-wide level, we isolated total RNAs from the *C. neoformans* JEC21 strain. After eliminating ribosome RNAs (rRNAs) and treating with RNase R, the total RNA was utilized to construct libraries for deep sequencing by the Illumina HighSeq 2500 platform. The sequencing data reached 6.26 Giga nucleotides (Gnt) raw bases in total, covering 41.70 million paired-end individual reads sized above 150 nt. After trimming adaptors and filtering low-quality reads, we obtained 39.28 million clean reads (Table 1). 

Clean reads were then mapped to the *C. neoformans* JEC21 genome by BWA software. The mapped reads were input to CIRI, a published circRNA identifier, to identify the candidates of circRNAs. To reduce false-positive candidates, the circRNAs that had more than one back-splicing junction read were considered. After a two-step filtration, 73 individual circRNAs containing high-confident back-splicing junctions were obtained. The number of reads for the 73 unique circRNAs was counted to 820. Only 20 of the 73 circRNAs (27.4%) had more than four back-splicing junction reads. The 10 with the highest junction reads are listed (Table 2) and detailed information on all predicted circRNAs is available (Supplementary Table S2). The above data show that the absolute number of unique cryptococcal circRNAs is low compared to that of circRNAs in higher eukaryotes, such as animals or plants. Specifically, researchers have detected 3001 circRNAs from human cells [27] and 5372 circRNAs from soybeans [28]. However, when referring to the relative expression levels using the ratio of circRNAs number to genome size (Mb), the results changed in which the relative expression of *C. neoformans* circRNAs (~3.74) is much higher than that of human (~1.00), but a little lower than soybean (~4.88).

**Figure 1:**
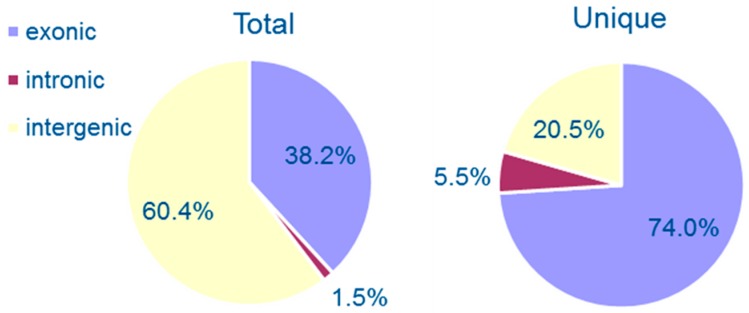
Percentages of three groups of circular RNAs. The circRNAs were classified as exonic, intronic, and intergenic according to the back-splicing junction position on chromosomes. Total circRNAs, calculated as back-splicing junction reads, are shown in the left panel, while unique circRNAs are shown in the right panel.

We sorted the unique circRNAs into three groups according to the positioning of their two ends on chromosomes (exonic, intronic, and intergenic regions). Among them, 54 (73.97%) of the 73 circRNAs were generated from exons of protein-coding open reading frames (ORFs) and 15 (20.55%) were intergenic circRNAs. Only four (5.48%) had intronic junctions. Besides unique circRNAs, we also calculated the total reads of each type of circRNA. Our data show that 38.17, 1.46, and 60.37% of the total 820 reads were distributed to exonic, intronic, and intergenic circRNAs, respectively (Figure 1). However, exons, introns and intergenic sequences occupy 54.14, 11.97, and 33.89%, respectively, of the whole *C. neoformans* genome [20]. Thus, these results reveal that intergenic circRNAs have higher mean reads than exonic circRNAs, although the latter consists of the majority of unique circRNAs. 

### 3.2. Properties of Cryptococcal Circular RNAs

**Figure 2:**
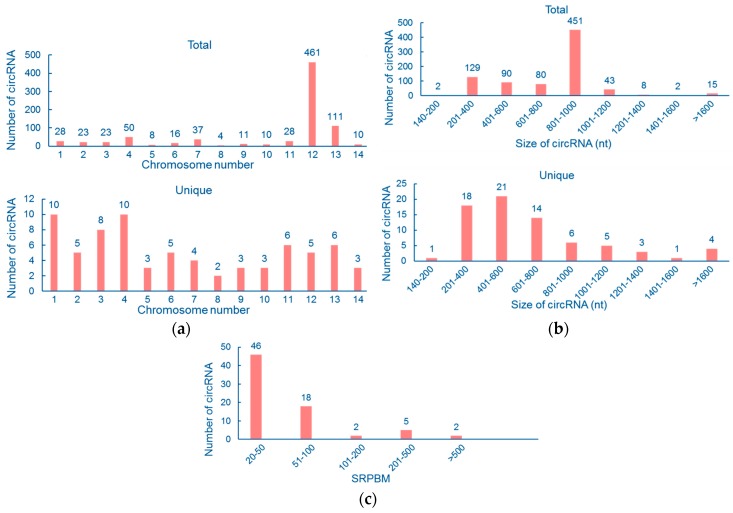
Counting calculations of circRNAs in *C. neoformans*. (**a**) Chromosome distribution of unique circRNAs (bottom panel) and total reads (upper panel). (**b**) Size distribution of unique circRNAs (bottom panel) and total reads (upper panel). (**c**) Expression level distribution of unique circRNAs. SRPBM: spliced reads per billion mapping.

In order to determine the properties of cryptococcal circRNAs, we performed a set of counting calculations for unique and total circRNAs respectively. Firstly, chromosomal distribution for unique and total circRNAs was examined. According to our analysis, 461 total reads were located on chromosome 12. The reason is simple: the highest-expressed circRNA, circ12:174494-175325 (410 reads), was found on Chr12. Correspondingly, chromosome 8 contains the least amount of total back-splicing junction reads, which is only four (Figure 2a, upper panel). The distribution of unique circRNAs among the 14 chromosomes is also displayed in Figure 2a, bottom panel. We found that chromosome 8 contains the least amount of unique circRNAs, two, and chromosomes 1 and 4 contain 10 each.

Secondly, we examined the size distribution of cryptococcal circRNAs. For unique circRNAs, the length was mostly (72.60%) between 201 and 800 nt (Figure 2b). Only a few unique circRNAs were <200 nt (1.37%) or >1400 nt (6.85%). As for total reads, the length concentrated on 801–1000 nt as the size of circ12:174494-175325, which possesses the most reads, is 831 nt. Also, only two reads in total circRNAs were found <200 nt (0.24%, totally) and 17 were >1400 nt (2.07%, totally). Finally, we normalized the expression of each unique circRNA to spliced reads per billion mapping (SRPBM), in order to analyze their expressional features. SRPBM of most circRNAs (63.01%) was <50, while only two circRNAs (2.74%) had SRPBM >500 (Figure 2c). 

### 3.3. Functional Analysis of circRNA Host Genes 

**Figure 3:**
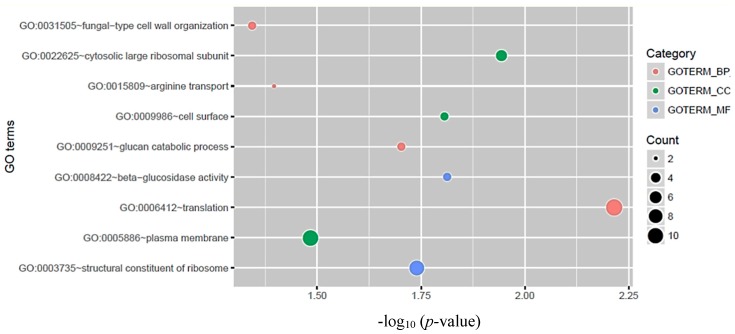
Gene Ontology (GO) category analysis of circRNA host genes in *C. neoformans*. GO terms with the threshold of *p*-value < 0.05 are listed. GO terms were classified in three categories: Biological process (BP), cellular component (CC), and molecular function (MF).

To investigate the function of circRNA host genes, we performed GO analysis and KEGG pathway analysis. GO analysis suggested that circRNA host genes mainly encode proteins of the large ribosomal subunit, cell surface proteins, and plasma membrane proteins (*p*-value < 0.05). For GO molecular function analysis, circRNA host genes were associated with beta-glucosidase activity and structural constituents of ribosome (*p*-value < 0.05). For the GO biological process, those genes were enriched in translation, glucan catabolic process, arginine transport, and fungal cell wall organization (*p*-value < 0.05) (Figure 3). As for distribution in KEGG pathways, the results show that circRNA host genes were significantly (*p*-value < 0.05) enriched in two pathways: The ribosome biogenesis and starch-sucrose metabolism pathways (Table 3).

### 3.4. Small Guanosine Triphosphatase-Encoding Orthologs Are Conserved circRNA Hosts

**Figure 4:**
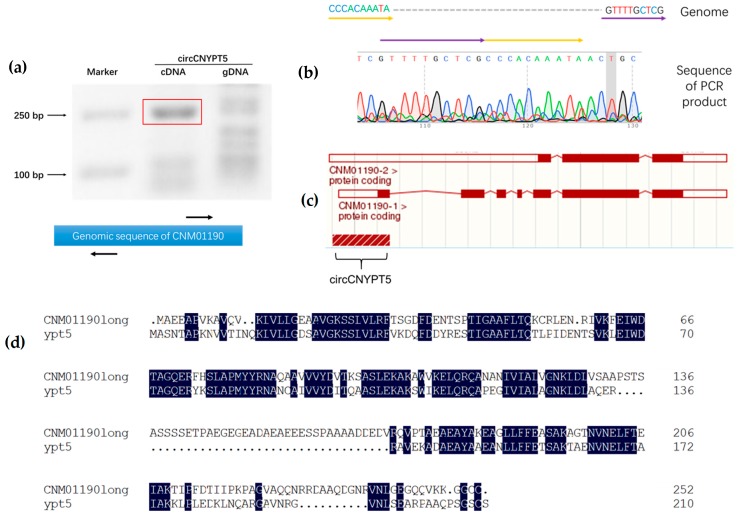
Small GTPase-encoding genes are conserved in circRNA origination among yeasts. (**a**) The upper panel shows that circCNYPT5 can be amplified using outward polymerase chain reactions (PCRs). By contrast, PCR using a genomic DNA template was unable to produce a corresponding band at the same level. Primers in the amplification (black arrows) are shown in the bottom panel. (**b**) Sanger sequencing confirmed the back-splicing junction in PCR products. The 5′ (yellow arrow) and 3′ (purple arrow) were found connected according to sequencing data. (**c**) circCNYPT5 is derived from the 5′ region of *CNM01190*, which has two splice variants. (**d**) Multiple alignments of amino acid sequences showed a high identity between proteins encoded by *CNM01190* in *C. neoformans* and *YPT5* in *Schizosaccharomyces pombe*. Identical amino acids between the two proteins are highlighted in dark blue.

In order to validate the existence of circRNAs, we performed verification by PCR amplification with a pair of outward-designed primers and a cDNA template. In total, primers and PCR reaction systems for six potential circRNAs were designed, including the circ13:359406-359654 whose expression was the highest among all exonic circRNAs (Supplementary Table S1). Consequently, only two specific bands were obtained, respectively, for circ13:359406-359654 and circ7:1027082-1027487. To further confirm the results, we successfully got the PCR band with expected length (248 bp) for circ13:359406-359654 (Figure 4a). Not surprisingly, we failed to observe a corresponding band at the exact level in the control reaction, in which genomic DNA was used as a template. Subsequent Sanger sequencing confirmed that it contained the predicted back-splicing junction site (Figure 4b). 

According to genomic distribution data, circ13:359406-359654 was derived from the 5′ end of the gene *CNM01190* (National Center for Biotechnology Information [NCBI] ID: 3255052). By virtue of splicing information stored in the Ensemble Fungi database, we found that the gene *CNM01190* has two splice variants, which encode either a short version (174 aminoacides (aa)) or a long version (252 aa) of protein (Figure 4c). Through a conserved domain basic local alignment search tool (BLAST) search [29], we found that the long variant product encoded by *CNM01190* belongs to the Rab-related GTPase family, which is exemplified by *YPT5* in *Schizosaccharomyces pombe*. Given this, we named the cryptococcal circRNA circCNYPT5. We then used Clustal Omega to compare amino acid sequences of *YPT5* and *CNM01190* long version and found that their identity rate was as high as 57.62% (Figure 4d). Surprisingly, *YPT5* has also been reported to generate circRNAs in *S. pombe*, residing at the exonic regions instead of the 5′-untranslated region (5’-UTR) [17,30]. Therefore, we also performed DNA sequence alignments between the two homologous genes with the BLAST algorithm available at the NCBI website, but no significant similarity was found. In general, our data suggest a conservation of circRNA host genes in yeasts. 

### 3.5. DBR1 Expression Level Is Negatively Associated with circDPEPS but not circCNYPT5

**Figure 5:**
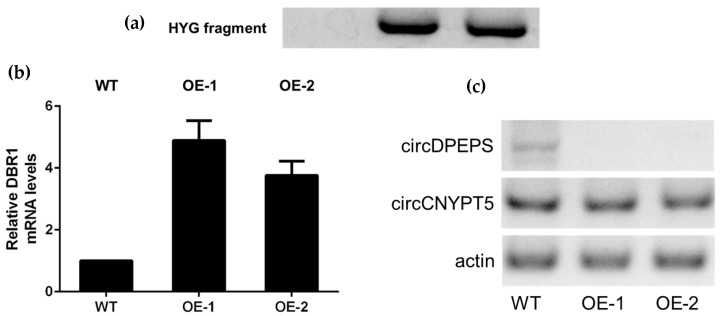
Overexpression (OE) of *DBR1* has a diverse influence on circDPEPS and circCNYPT5. (**a**) PCR was performed to confirm that *DBR1* overexpression of plasmid pBS-HYG-DBR1 was transformed into two transformants, OE-1 and OE-2 (lane 2 and lane 3). JEC21 (wild-type, WT) was used as control. (**b**) Quantitative reverse-transcription (RT) PCR analysis showed that *DBR1* expression level increased up to 4.89- and 3.76-fold in OE-1 and OE-2 compared to WT. Error bars show standard error of the mean. (**c**) Semiquantitative RT-PCR analysis shows that the circDPEPS level declined significantly in the OE strains, while circCNYPT5 remained at a similar level in all three strains. Actin-encoding gene *ACT1* mRNA was used as an internal control.

According to the lariat-driven model, circRNAs are derived from exon-containing lariat precursors. Inhibiting Dbr1 protein, which degrades lariat, could lead to increased circRMA levels. The expression level of global circRNAs in the *DBR1Δ* mutant strain increased by three- to four-fold over the level in the wild-type (WT) *S. pombe* [31]. To investigate the function of *DBR1* in circRNA regulation in *C. neoformans*, we tried to knock out the counterpart *DBR1* gene in *C. neoformans* JEC21 with the latest clustered regularly interspaced short palindromic repeats (CRISPR)-Cas9 editing system [32,33]. Unfortunately, we failed to get any *DBR1* knockout strain, which implies that the *DBR1* gene might be essential in serotype D strains. As a solution, we overexpressed the *DBR1* gene in JEC21. The overexpression (OE) vector pBS-HYG-DBR1 was detected in transformants OE-1 and OE-2, but not WT, through amplification of a hygromycin-resistant fragment (Figure 5a). Quantitative reverse-transcription (RT) PCR showed that *DBR1* mRNA increased up to 4.89- and 3.76-fold in OE-1 and OE-2, respectively, compared to WT (Figure 5b). 

We speculated that overexpression of *DBR1* could reduce circRNA levels in *C. neoformans*. Thus, we analyzed the expression levels of circCNYPT5 in OE-1, OE-2, and WT by semiquantitative RT-PCR. To our surprise, no significant difference in circCNYPT5 expression level was found between WT and overexpression strains according to the electrophoretogram (Olympus, Tokyo, Japan). On the other hand, another highly expressed circular RNA, circDPEPS, which is derived from the whole sequence of gene *CNG03660* (putatively encoding DNA polymerase epsilon p12 subunit, gene ID: 3258898), showed a dramatically decreased level in both overexpression strains (Figure 5c). One possible explanation for these divergent results is that circDPEPS might be processed through an exon-containing lariat precursor, while the generation of circCNYPT5 might rely on a lariat-independent mechanism. On the other hand, these two circRNAs might both be regulated by the *DBR1* gene which plays an important role upstream, but the different downstream action elements may contribute to the wildly different results, which requires further investigation.

## 4. Discussion

In the present study, we report the results of a genomewide screening for circRNAs in the basidiomycetous yeast *C. neoformans* serotype D JEC21 using RNA-seq with bioinformatics analysis. A total of 73 unique circRNAs—including 54 exonic, 4 intronic, and 15 intergenic circRNAs—were identified by the CIRI algorithm. Considering the number of exonic circRNAs and intronic circRNAs, we got 58 predicted circRNA host genes, which was near the number obtained from *S. pombe* (42 genes) [30], but notably less than higher eukaryotes [27,28,34]. The reason might be that different biosynthesis paths are adopted in various organisms. For instance, neural circular RNAs were generated in a spliceosome-dependent manner [35], whereas lariat precursors, which are byproducts in an exon-skipping event, are required for the production of circRNAs in *S. pombe* [17]. 

Furthermore, we conducted an analysis of the functions of circRNA host genes by GO and KEGG pathways. According to the top-rank rule in GO annotation, we found that circRNA host genes are enriched in encoding structural proteins of the ribosome, plasma membrane, and cell wall, all of which are important in routine growth of the yeast. In addition, we found that the ribosome and starch–sucrose metabolism pathways are the most enriched pathways in the KEGG analysis. However, the relationship between circRNAs and their host genes still remains elusive due to the limitations of the mutagenesis strategy in the study of circRNAs. CircRNA biosynthesis can compete with pre-mRNA splicing in some cases [36]. On the other hand, some circular intronic RNAs (ciRNAs), such as Ci-ankrd52, are able to promote the transcription of corresponding genes [37]. Additionally, circRNAs in plants like rice and tomato usually exhibit developmental specificity [38,39], while soybean circRNAs show mainly tissue specificity [28]. 

Interestingly, we found that Rab GTPase-encoding orthologs are conserved circRNA hosts in *C. neoformans* and *S. pombe*. In fact, previous studies reported GTPase-derived circRNAs in protists [30] and humans [40]. Given the low occurrence of unique circRNAs in yeasts, these results may have meaningful implication as to their biogenesis and the function of circRNAs. In our yeast, the host gene, *CNM01190* (NCBI ID: 3255052), has two alternative splicing products that putatively encode 174-aa and 252-aa proteins separately. The circCNYPT5 was derived from the 5′-UTR region of the gene and the first exon in the longer variant. Whether the formation of circCNYPT5 is associated with the alternative splicing process is an intriguing question. The long version of *CNM01190* encodes a member of the small GTPase family. Many of the members of the family have been shown to play vital roles in pathogenesis, thermotolerance, mating, and septin localization in *C. neoformans* [41,42,43]. Thus, circCNYPT5 that originated from the 5′-UTR region of the gene could presumably act as a profound regulator of the transcription of the *YPT5* gene. Biogenesis of circRNAs within the conserved host ortholog genes across the eukaryotic domain of life raises a question for further investigation. 

Finally, we showed that the RNA lariat debranching enzyme (*DBR1*) has variable influence on different circRNA expression in *C. neoformans*. *DBR1* was demonstrated to play key roles in circRNA biogenesis by the lariat-driven pathway in fission yeast [17]. Whether cryptococcal *DBR1* mediates circRNAs biosynthesis in a fission yeast style is still unknown. We failed to knock out *DBR1* in the *C. neoformans* JEC21 serotype D strain, although it could be deleted in the serotype A H99 strain [44]. This phenomenon suggests diverse functions of this gene. Overexpression of *DBR1* in the JEC21 strain decreased circDPEPS levels but had no effect on circCNYPT5 levels. We also attempted to detect other potential circRNAs with PCR amplification but failed, maybe due to the background disturbance from rRNA and linear RNA which were removed from the RNA samples in the RNA-seq. Our data indicate that circCNYPT5 is not processed through an exon-containing lariat precursor. As to the biogenesis of circCNYPT5, the intron-driven model may not be applicable in this case, as no intronic secondary structure was predictable by Mfold software [45]. Whether circCNYPT5 is produced through a spliceosome-dependent path or a protein factors-associated path needs to be investigated. Consistent with our research, a recent RNA-seq study revealed an approximately three- to four-fold increase in circRNAs when *DBR1* was mutated in *S. pombe* [31]. However, researchers also found that the effect of *DBR1* deletion on particular genes seems to have statistical significance [17]. As for circDPEPS, it might be produced through a classic lariat-precursor pathway. However, there is still the possibility that this circRNA derives from other pathways. For instance, the *DBR1* gene may have an indirect influence on circDPEPS expression due to the multiple functions of *DBR1*. It is noteworthy that, according to recent work on *C. neoformans* serotype A H99, *DBR1* is indispensable in the biosynthesis of some transposon-derived small interference RNAs (siRNAs) [44]. In sum, the exact cryptococcal circRNA biosynthesis pathway, along with the comprehensive function of *DBR1* in *C. neoformans*, may need further investigation. 

## 5. Conclusions

In the present study, we identified 73 unique circular RNAs from basidiomycetous yeast *C. neoformans* using RNA-seq and bioinformatics tools such as CIRI. The function of circRNA host genes enriches in primary metabolism, especially translation and carbohydrate metabolism. In addition, we found that the small GTPase ortholog genes are conserved hosts of circRNAs among eukaryotic organisms. Primary analysis revealed that the cryptococcal *DBR1* gene has a differential impact on the generation of different circRNAs. Our study on the identification of circRNAs opens an avenue to understanding their biological function in the pathogenesis of this pathogen. 

## Figures and Tables

**Table 1:** RNA-sequencing data of *Cryptococcus neoformans* JEC1.

Sample Name	Raw Reads	Filtered Reads	Raw Base	Filtered Base	Q20 (%) ^1^
Cn JEC21	41,703,834	39,280,024	6.26 Gnt	6.16 Gnt	98.52

^1^ Q20 refers to the percentage of nucleotides with Phred quality score > 20, which means base accuracy is 99%. Gnt: Giga nucleotides.

**Table 2:** Detailed information on the 10 circRNAs with the highest back-splicing reads.

circRNA ID	Chr	RNA Size	circRNA Start Loci	CircRNA End Loci	Junction Reads ^1^
12:174494-175325	12	831	174494	175325	410
13:359406-359654	13	248	359406	359654	67
7:1027082-1027487	7	405	1027082	1027487	29
13:603431-604144	13	713	603431	604144	28
4:1303545-1304160	4	615	1303545	1304160	24
12:174265-175325	12	1060	174265	175325	23
12:174461-175325	12	864	174461	175325	22
11:62574-63095	11	521	62574	63095	11
13:89597-90783	13	1186	89597	90783	8
2:661847-663003	2	1156	661847	663003	7

^1^ Junction reads means counts of back-splicing reads. Chr: Chromosome.

**Table 3:** Kyoto Encyclopedia of Genes and Genomes pathway enrichment analysis of circRNA-host genes in *C. neoformans*.

Pathway ID ^1^	Description	Gene Count	*p*-Value
cne03010	Ribosome	6	0.023
cne00500	Starch and sucrose metabolism	3	0.031

^1^ Pathway with the threshold of *p*-value < 0.05 is listed.

## Supplementary Materials

The following are available online at http://www.mdpi.com/2073-4425/9/3/118/s1. Table S1: Primers used in this study. Table S2: List of all predicted circRNAs in C. neoformans JEC21.
